# Supplementary material for: SyNDI: synchronous network data integration framework
Source: BMC Bioinformatics. 2018 Nov 6;19:403. doi: 10.1186/s12859-018-2426-5 (PMC6219086; doi:10.1186/s12859-018-2426-5)
Supplement: Supplementary file 5 — Files of the example presented in Additional file 4. This additional file is a zip package that contains all files used in Additional file 4. Detailed descriptions of each of these files are presented in a README file included in this zip package. (GZ 30411 kb) [file 12859_2018_2426_MOESM5_ESM.gz › SyNDITools/Meme2Fimo/meme_4.9.0_4.tar.gz!/meme_4.9.0/website/html/documentation.html]

MEME Suite - Web-based Tools Documentation


| MEME Suite The MEME Suite web application requires the use of JavaScript Javascript doesn't seem to be available on your browser. Publications \| Release Notes \| FAQ \| Command Line Versions; Motif Discovery \| \| \|; DREME \| Manual \| Tutorial \| Sample Output; GLAM2 \| Manual \| Tutorial \| Sample Output; MEME \| Manual \| Tutorial \| Sample Output; MEME-ChIP \| Manual \| \| Sample Output; Motif Search \| \| \|; FIMO \| Manual \| Tutorial \| Sample Output; GLAM2SCAN \| Manual \| Tutorial \| Sample Output; MAST \| Manual \| Tutorial \| Sample Output; Motif Enrichment Analysis \| \| \|; SpaMo \| Manual \| Tutorial \| Sample Output; CentriMo \| Manual \| Tutorial \| Sample Output; Motif Cluster Search \| \| \|; MCAST \| Manual \| Tutorial \| Sample Output; Motif Comparison \| \| \|; TOMTOM \| Manual \| Tutorial \| Sample Output; Motif Function Analysis \| \| \|; GOMO \| Manual \| Tutorial \| Sample Output |
